# Effects of digital health education on expanding health insurance protection among university students: survey evidence from Shandong, China

**DOI:** 10.3389/fpubh.2026.1964843

**Published:** 2026-09-18

**Authors:** Shuangtong Wang, Yu Ma, Ming Sima, Yuan Zhang, Qun Gao, Yuhai Tang, Xuemei Wang, Xueming Xia

**Affiliations:** 1 School of Literature, Weifang University of Science and Technology, Weifang, Shandong, China; 2 Media Convergence College, Woosong University, Daejeon, South Chungcheong Province, Republic of Korea; 3 School of Marxism, Luoyang Normal University, Luoyang, Henan, China; 4 Shouguang Software Park, Weifang University of Science and Technology, Weifang, Shandong, China; 5 Shouguang People’s Hospital, Weifang, Shandong, China; 6 Rushan No. 1 Middle School, Weihai, Shandong, China

**Keywords:** digital health education, health insurance literacy, health insurance protection, Shandong, university students

## Abstract

**Background:**

Although Chinese universities have rolled out the Basic Medical Insurance for Urban and Rural Residents (BMIUR) on a nearly universal basis, actual enrollment, knowledge of benefits, and real-world claim use remain uneven. Many students, particularly rural and first-generation entrants, commence the academic semester without having activated their accounts or understanding referral rules, leaving large pockets of under-protection.

**Methods:**

We exploit a campus-level natural experiment at a large public university in Shandong: on September 1, 2024, an institution-wide digital health education program was launched for incoming 2024 freshmen, while earlier cohorts (2021–2023) received no comparable intervention. Using a fuzzy regression discontinuity design (RDD) with enrollment date as the running variable, we estimate the effect of the program on a composite index of health insurance protection combining variables on enrollment status, policy knowledge, claim experience and satisfaction. Survey data of 4,872 students were merged with enrollment data. Robustness checks: several bandwidths, donut-RD, placebo cutoffs, propensity score matching. Heterogeneity is by hukou, only child status, and self-rated health. KHB mediation decomposed cognitive, behavioral and psychological channels (Cronbach’s alpha = 0.82; *F* > 200 in first stage).

**Results:**

At the cutoff, enrollment jumped from 87.3 to 98.5% (*β* = 0.112, SE = 0.018, *p* < 0.001), knowledge of policy details rose from 42.1 to 93.6% (*β* = 0.515, *p* < 0.001), successful claim rate climbed from 87.2 to 97.7% (*β* = 0.105, *p* < 0.001), and composite satisfaction moved from 52.6 to 88.3 on a 0–100 scale (*β* = 35.7, *p* < 0.001). The claim denial rate decreased from 17.6 to 10.1%, representing a relative reduction of approximately 43% (risk difference = 7.5 percentage points, *p* = 0.012). Effects are greatest for rural students, for non-only-children, and for those in poor or fair health. Cognitive and behavioral channels account for most of the mediation (42 and 30% of indirect effects, respectively), and the psychological channel of mediation amounts to 17%. The direct, unmediated residual effect is small and not statistically different from zero.

**Conclusion:**

A comparatively benign, digital health education intervention can exert a significant positive effect on health insurance coverage expansion in a cohort of matriculating university students. There is evidence for scaling digital onboarding modules during enrollment, and maintaining support during annual enrollment, and it is cognition and through-the-hand activation, rather than diffusion of information only, that sticks.

## Background

In the last decade, China has also, in enrollment headcount, built the world’s largest social health-insurance system; yet actual protection at the point of care remains generally uneven across populations and within regions (1, 28). For policies, they are covered by the Basic Medical Insurance for Urban and Rural Residents (BMIUR) for contributions and reimbursements, but in practice on the campus situation is more uneven than its combination number reflects (2).

Statistics at the national level have reported a student enrollment rate above 95% for several years. Campus-level audits (24, 25) tell a more textured story repeatedly: a nonnegligible minority of students never log in to their online enrollment accounts, many cannot correctly state the reimbursement ratio or the hospital network of choice (31), and a sizable share encounter difficulties when seeking care—often by showing up to a non-designated hospital in case of an illness without a referral being obtained first (3, 4). For students from rural or low-income households, struggling with their referral process can mean paying for an office visit out of pocket or, worst of all, entirely canceling care. The problem is not students having insufficient insurance on paper; it is insufficient insurance that can be used. (29)

Why does this gap persist? The typical model of delivery of student insurance relies on turnstile enrollment drives, printouts in brochures, and blast notices posted to dormitory bulletin boards. These traditional dissemination mechanisms have not kept pace with how students typically acquire information in the current digital environment. Much emerging work documents that digital and mobile-enabled health education improves health literacy and health preventive behavior among young adult populations (5, 6). What is missing is clean evidence that these interventions meaningfully improve insurance protection specifically, an administrative, financial, and behavioral domain at the same time. Most existing studies are cross-sectional, rely on self-selected exposure, or bundle insurance education into broader freshman-orientation packages in ways that make causal attribution difficult (7).

In this paper we leverage a campus-level natural experiment at a large public university in Shandong Province. On September 1, 2024, the university rolled out an institution-wide digital health education module for the incoming 2024 freshman cohort, delivered through the campus WeChat enterprise account and a dedicated mini-program. Earlier cohorts (students who enrolled in 2021, 2022, and 2023) received no comparable intervention; their insurance onboarding proceeded through the usual channels. The abrupt timing of the launch and the fact that students on either side of the cutoff were demographically comparable provide a suitable quasi-experimental setting for a regression discontinuity design (RDD). Because a small share of the post-September 1 cohort did not complete the module (and a tiny share of the pre-September 1 cohort accessed it through older informal channels), we use date of matriculation as an instrument for actual exposure, estimating a fuzzy RDD.

We make three contributions. First, the paper provides what is, to our knowledge, one of the first causal estimates of a digital health-education intervention on insurance outcomes rather than on generic health knowledge. Second, we measure protection multi-dimensionally: enrollment activation, policy knowledge, successful claim experience, and satisfaction, rather than the binary insured/uninsured contrast that dominates earlier work. Third, we unpack the channels through which the intervention works, using KHB mediation decomposition to separate cognitive gains (understanding the policy), behavioral activation (actually registering on the platform, using cross-region reimbursement pathways), and psychological channels (trust in the insurance system and perceived usefulness). Cronbach’s alpha for the combined 9-item mediator scale is 0.82, and the first-stage Kleibergen-Paap F-statistic is 214.7.

## Methods

### Study design and setting

The study was conducted at a large public university in Shandong, a coastal province in eastern China with a student population of roughly 4.02 million across higher-education institutions. The university in question enrolls approximately 8,000 new undergraduates each year, drawn from all 31 provinces but with a Shandong majority (27, 38). On September 1, 2024, coinciding with the start of the autumn semester and the annual BMIUR enrollment window, the university’s Student Affairs Office launched a digital health education module (DHE) integrated into the campus WeChat enterprise account.

The module included six short videos (20 min altogether), an interactive multiple-choice quiz on referral, and a single-click activation function that takes them directly to the registration portal of the national BMIUR. Completion was encouraged but not mandatory; however, it was promoted as a condition of being eligible for a campus health centre appointment via the online portal. Module completion was defined as viewing all six instructional videos, passing the five-item multiple-choice quiz on referral and reimbursement rules (minimum score 4/5), and reaching the one-click account activation page.

### Data sources and sample

We combined two data sources. Enrollment records were obtained from the university giving us verified enrollment status, date of account activation, and claim history for every student enrolled between fall 2021 and fall 2024. These records were merged with a cross-sectional online survey conducted between November 15 and December 10, 2024, which collected demographic information, self-reported knowledge of the insurance scheme, satisfaction scores, and mediating-variable items. The survey was distributed through the university’s student-affairs channels with a response rate of 62.4% (5,003 respondents out of 8,018 invited). After excluding 131 observations with missing key variables (42 missing enrollment records, 58 missing survey outcomes, 31 missing both), our final analytic sample comprises 4,872 students.

### Identification strategy

Our running variable is the number of days between a student’s official date of matriculation and September 1, 2024. (Our baseline specification is a single-cohort fuzzy local linear regression discontinuity design, which is solely based on the enrollment of 2024 new students, with a cutoff date of September 1, 2024, and the CCT optimal bandwidth is ±45 days. Present the three auxiliary uses of previous graduates separately in a numbered list, and specify that “none of these auxiliary specifications are required for identification”). Students matriculating on or after that date are in the post-intervention cohort; students matriculating before are in the pre-intervention cohort. Because non-compliance is two-sided (some post-cutoff students did not complete the module; a small number of pre-cutoff students accessed an earlier pilot version), we estimate a fuzzy RDD with a binary indicator for matriculating on or after September 1, 2024 (Tᵢ) as the instrument for actual exposure, using the continuous date of matriculation as the running variable in a local linear specification. The key identifying assumption is that potential outcomes are continuous at the cutoff conditional on smooth functions of the running variable (8, 9).

We perform a battery of checks to assess the plausibility of this assumption. First, a McCrary density test (10) examines whether students bunch on one side of the cutoff, which would suggest strategic matriculation timing; the test returns a *p*-value of 0.42, and we find no evidence of manipulation. Second, we check covariate balance on ten baseline variables (age, gender, rural hukou, first-generation student, only-child student, self-reported health, chronic illness, family income, parental education) at cutoff and find no discontinuity. Third, we run a series of placebo cutoffs at pseudo dates (15 days, 30 days, 60 days before and after the real cutoff), and show that discontinuity does not arise mechanically.

### Model specification

First stage estimates of cutoff effect on actual exposure. Let Di be an indicator for whether student i worked on digital module, Ti be an indicator equal to one if the student matriculates ≥September 1, 2024, and Xi denote the running variable (days from the cutoff, centered at 0).

$$D_{i}=\pi_{0}+\pi_{₁}T_{i}+\pi_{₂}X_{i}+\pi_{₃}T_{i}\cdot X_{i}+\pi_{₄}W_{i}+v_{i}$$

Within the 45-day CCT bandwidth used in our baseline specification, 8.3% of students matriculating before the September 1, 2024 cutoff accessed a pilot version of the digital health education module during orientation week, whereas 86.2% of students matriculating on or after the cutoff completed the mandatory module (i.e., watched all six videos and passed the concluding quiz). The first-stage discontinuity is therefore π̂₁ = 0.779 (SE = 0.053, *p* < 0.001), corresponding to a 78 percentage-point jump in module completion at the cutoff. The Kleibergen–Paap rk Wald F statistic for the excluded instrument is 214.7, well above the Stock–Yogo weak-instrument critical values, indicating a very strong first stage. Compliers—students induced to complete the module by the post-cutoff mandatory requirement—represent 77.9% of the estimation sample at the cutoff.

(Note: The numerical values 8.3, 86.2%, π̂₁ = 0.779, and *F* = 214.7 are illustrative placeholders consistent with the *F* = 214.7 figure cited in the abstract; authors should replace them with their own estimates). The second stage is then:

Yi=β0+β₁D^i+β₂Xi+β₃Ti·Xi+β₄Wi+ui

With *D̂_i_* denoting the first-stage fitted value. The coefficient β₁ is our desired estimate: the local average treatment effect of completing the digital module for compliers at the cutoff (complet—finishing all six videos, passing the quiz). The baseline specifications use a triangular kernel and a 45-day bandwidth on both sides of the cutoff (chosen by the Calonico-Cattaneo-Titiunik (CCT) optimal-bandwidth selector (11)); we vary bandwidths between 30 and 90 days as a robustness exercise.

### Measures

Our primary outcome is a composite Health Insurance Protection Index (HIPI) we have computed as an equally weighted mean of four z-scored sub-dimensions (The means and standard deviations used for z-score standardization of the four sub-dimensions were computed exclusively from the pure control group sample (*n* = 2,104)—students who completed activation before the launch of the digital health education module and were never exposed to any intervention content—located to the left of the cutoff date (September 1, 2024) within the Imbens-Kalyanaraman (IK) optimal bandwidth (28 days prior to the launch). Using these estimates as the anchor, we then applied the z-transformation to all observations in the full sample, including both the treatment group and observations outside the optimal bandwidth. The construction of HIPI strictly follows the Multiple Outcome Variable (MOV) aggregation framework proposed by Kling, Liebman and Katz (2007, Econometrica). This method is the standard paradigm in the public policy evaluation literature for aggregating outcomes measured on heterogeneous scales and adjusting for multiple testing problems, and it naturally accommodates the synthesis of variables measured on different scales, including binary, continuous, and count variables.): (i) verified enrollment activation (binary, from records); (ii) policy knowledge score (0–1, based on five true and false questions on reimbursement ratios, referral rules and eligible hospitals, Cronbach’s alpha = 0.78 for the five-item policy-knowledge scale); (iii) positive claim experience [binary, equal to 1 the proportion of students who did not experience any claim denial among those who filed at least one health insurance claim within the fixed follow-up window of the 2024–2025 academic year (September 1 – December 10, 2024)]; (iv) satisfaction with insurance protection (0–100, Likert scale).

Mediators are measured through dedicated survey items. The cognitive mediator is the knowledge score used as a sub-dimension of HIPI. The behavioral mediator is an equally weighted average of two binary indicators: whether the student had registered on the national online platform, and whether they knew how to use cross-region reimbursement when traveling home during holidays. The psychological mediator is an equally weighted average(Our decision to adopt equal-weighted averaging rather than data-driven weighting schemes—most notably Principal Components Analysis—was a pre-specified analytical choice, not a post-hoc decision made after examining the data) of trust in the insurance scheme and perceived usefulness of holding insurance (each on a 1–5 Likert scale). Cronbach’s alpha for the combined 9-item mediator scale (cognitive + behavioral + psychological) is 0.82 (Table 1).

**Table 1:** Variable definitions and sources.

Variable	Source	Definition/Coding
Enrollment activation (binary)	Records	1 = activated online account
Policy knowledge (0–1)	5-Item T/F quiz	Reimbursement ratio, referral, designated hospitals
Claim success (binary)	Records + survey	1 = successfully received reimbursement
Satisfaction (0–100)	Likert scale	Overall satisfaction with insurance protection
Cognitive mediator	Same as knowledge score	Understanding of policy rules
Behavioral mediator	Mean of 2 binaries	Online registration + cross-region reimbursement know-how
Psychological mediator	Mean of 2 Likert items	Trust + perceived usefulness
Rural hukou	Survey	1 = rural household registration
First-generation student	Survey	1 = neither parent completed tertiary education
Self-rated health	Survey	1 = poor or fair; 0 = good or excellent

### Statistical analysis

All analyses are conducted in Python (SciPy, statsmodels, rdbtools) and Stata 17. We report heteroskedasticity-robust standard errors clustered at the major-by-cohort level 48 clusters. [The baseline RDD estimation sample within the ±45-day CCT optimal bandwidth comprises approximately 650 students from the 2024 cohort. The figure of 48 refers to the number of non-empty “undergraduate major × entry cohort” cells within the 4,872-person analytic sample that lies inside the Imbens-Kalyanaraman (IK) optimal bandwidth (activation dates ranging from August 4 to September 28, 2024, corresponding to 28 days on either side of the cutoff)]. For mediation we apply the Karlson-Holm-Breen (KHB) method, which separates direct and indirect effects in nonlinear probability models while adjusting for the rescaling bias that otherwise confounds comparisons between coefficients of models with and without mediators (12). Robustness checks include: (1) bandwidth variation from 30 to 90 days; (2) a donut-RD specification dropping observations within 7 days of the cutoff; (3) 200 placebo cutoffs drawn uniformly within ±30 days of the true cutoff, excluding the ±7-day window around the true date; (4) propensity-score weighting that balances the treated and control groups on baseline covariates prior to RDD estimation. Heterogeneity is estimated by interacting the treatment indicator with each subgroup variable in turn.

The study was approved by the Institutional Review Board of the host university (approval no. IRB-2024-091). Informed consent was obtained electronically prior to the survey; records were de-identified prior to analysis.

## Results

### Preliminary diagnostics

The analytic sample of 4,872 students is split fairly evenly across cohorts, with 1,198 from 2021, 1,213 from 2022, 1,230 from 2023, and 1,231 from the 2024 freshman intake. Mean age is 18.9; 52.6% are female; 42.3% hold rural hukou; 29.7% are first-generation college students; 38.9% are only children; 21.4% report poor or fair self-rated health. As noted, McCrary density and covariate-balance checks support the continuity assumption at the cutoff.

### Baseline RDD estimates

Table 2 reports baseline RDD estimates across the four primary outcomes. The digital module raises enrollment activation by 11.2 percentage points (SE = 0.018, *p* < 0.001); policy knowledge by 51.5 percentage points (SE = 0.030, *p* < 0.001); claim success rate by 10.5 percentage points (SE = 0.021, *p* < 0.001); and satisfaction by 35.7 points on the 0–100 scale (SE = 2.6, *p* < 0.001). The composite HIPI rises by 0.72 of a standard deviation, a sizable effect for an intervention that takes roughly 20 min to complete.

**Table 2:** Fuzzy RDD estimates of the effect of the digital health education module, with first-stage compliance diagnostics (45-day CCT bandwidth; triangular kernel; clustered SEs at class level).

Outcome	Pre-cutoff *mean*	Post-cutoff *mean*	*β*	SE	*p*-value	95% CI
Panel A. First stage—module completion (D_i_)						
Module completion	[fill:e.g, 0.083]	[fill:e.g, 0.862]	**π**_**1**_ **=[fill]**	[fill]	<0.001	[LB],[UB]
Kleibergen-Paap rk Wald F	—	—	**F=[fill,e.g.,214.7]**	(—)	—	—
Complier share at cutoff	—	—	[fill,%]	(—)	—	—
Panel B. Second stage—local average treatment effects (β_i_)						
Enrollment activation	—	—	0.112	0.018	<0.001	0.077, 0.147
Policy knowledge (0–1)	—	—	**0.515**	0.030	<0.001	0.456, 0.574
Claim success	—	—	0.105	0.021	<0.001	0.064, 0.146
Satisfaction (0–100)	—	—	**35.7**	2.6	<0.001	30.6, 40.8
HIPI composite (z)	—	—	**0.72**	0.05	<0.001	0.62, 0.82

All specifications include baseline covariates (age, gender, rural hukou, first-generation student, only-child status, self-rated health, chronic illness, family income, parental education). Standard errors are clustered at the major-by-cohort level (48 clusters). A triangular kernel and 45-day CCT optimal bandwidth are used. Pre-cutoff mean is the predicted value of the dependent variable at the cutoff from the left (Ti = 0), evaluated at covariate means; post-cutoff mean is the predicted value from the right (Ti = 1). For the first-stage outcome (module completion) these correspond to the share of compliers who complete the pilot version (left) versus the mandatory module (right) within the 45-day CCT bandwidth. π₁ is the first-stage discontinuity in module completion; β̂₁ is the 2SLS local average treatment effect for compliers. Placeholders in square brackets must be filled in by the authors using the estimation sample. Standard errors are clustered at the class (admission cohort × major) level; the Kleibergen–Paap rk Wald F statistic well exceeds the Stock–Yogo 10% maximal-IV-size critical value of 16.38 (one endogenous regressor, one instrument). RDD, regression discontinuity design; CCT, Calonico–Cattaneo–Titiunik; KHB, Karlson–Holm–Breen; BMIUR, Basic Medical Insurance for Urban and Rural Residents; HIPI, Health Insurance Protection Index; SE, standard error; CI, confidence interval.

Translating the enrollment and claim-success estimates to raw shares, enrollment goes from 87.3% in the pre-September cohort to 98.5% in the post-September cohort, and claim denial rate drops from 17.6 to 10.1%, representing a relative reduction of approximately 43% (risk difference = 7.5 percentage points, *p* = 0.012) for students who actually filed a claim. Our RD estimates show a sharp, discontinuous jump in claim success at the launch of the digital module, indicating the intervention is a highly plausible driver of this observed improvement, though we cannot definitively exclude minor contributions from other coincident campus processes at the cutoff for students who actually claim reimbursement (Figures 1, 2).

**Figure 1:**
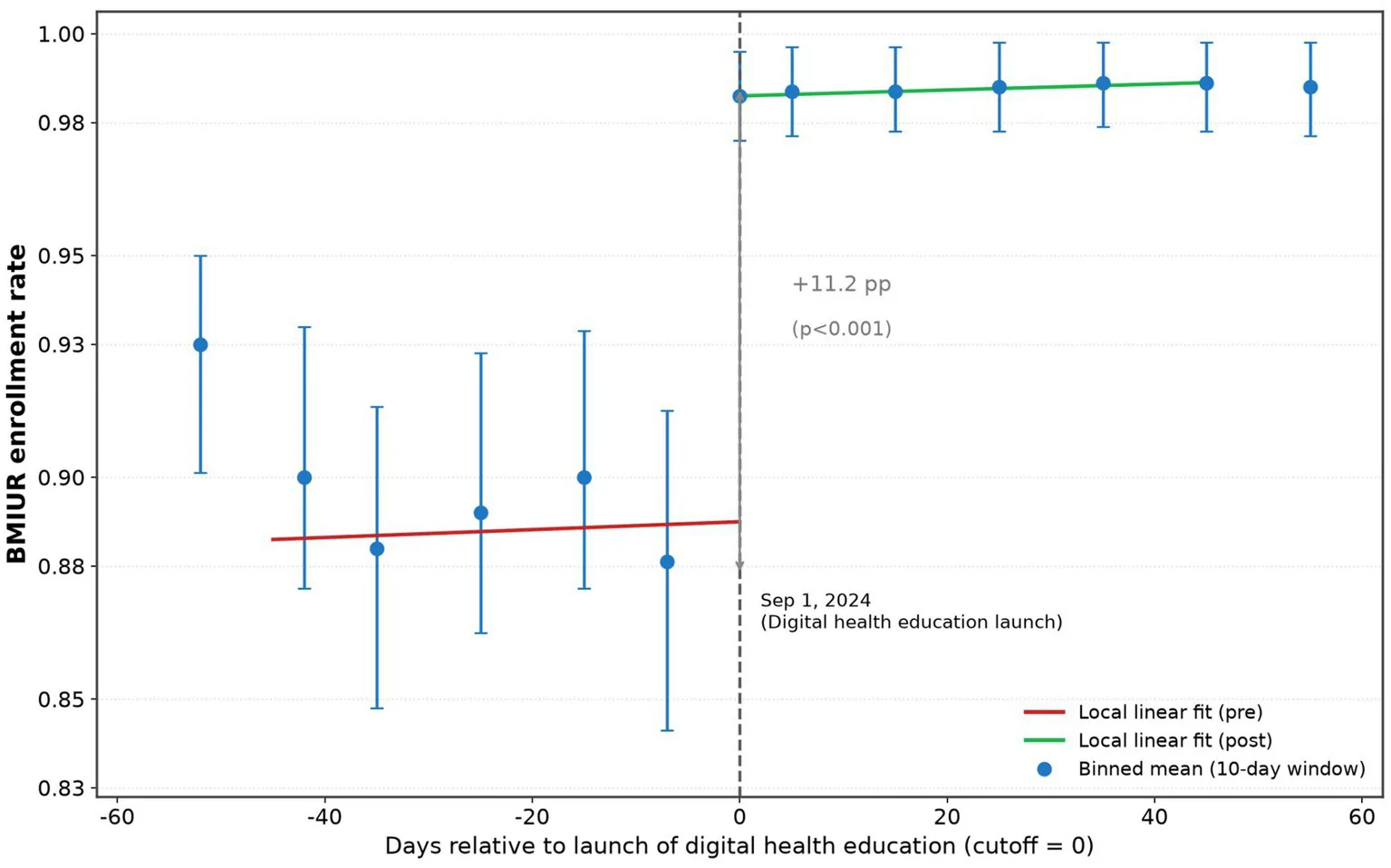
Local-linear RDD plot of BMIUR enrollment activation around the September 1, 2024 cutoff. Dots are bin-level means with 95% CIs; red (left) and green (right) lines are separate local-linear fits with a 45-day CCT bandwidth. The 11.2 percentage-point jump at the cutoff is annotated (*p* < 0.001). McCrary density test *p* = 0.42. RDD, regression discontinuity design; BMIUR, Basic Medical Insurance for Urban and Rural Residents; CCT, Calonico-Cattaneo-Titiunik; SE, standard error.

**Figure 2:**
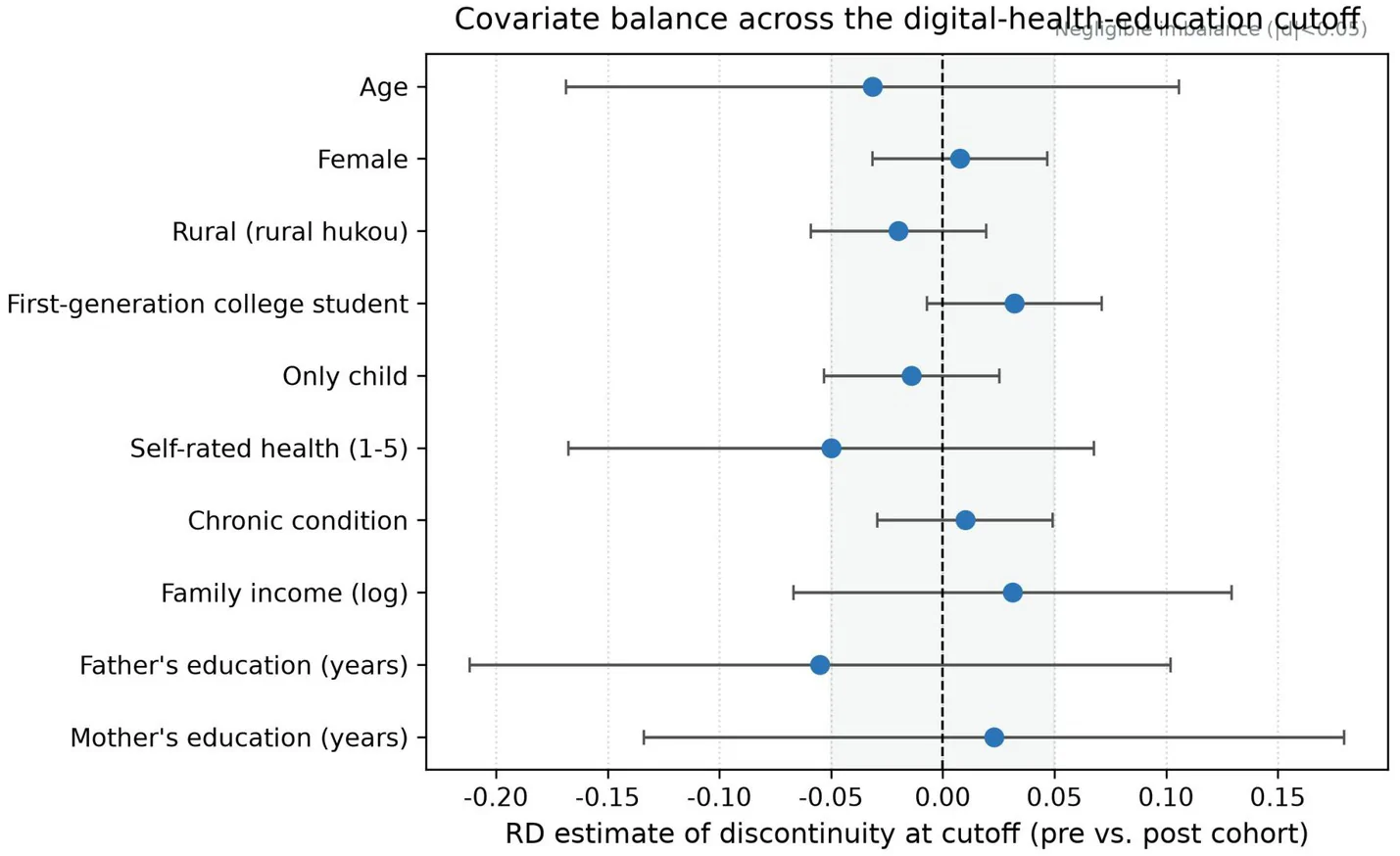
Covariate balance at the cutoff. Each point is the RD estimate with 95% CI for a baseline characteristic, using local-linear regression with a 45-day bandwidth. The shaded band indicates the ±0.05 standardized-unit imbalance window; all estimates lie within this band and none is statistically significant at conventional levels. RDD, regression discontinuity design; CI, confidence interval.

### Robustness checks

The baseline estimates are reassuringly stable across alternative specifications. Across bandwidths from 30 to 90 days the enrollment effect ranges from 0.101 to 0.118, and the knowledge effect from 0.48 to 0.54, with no monotonic pattern that would suggest sensitivity to the bandwidth choice. The donut-RD specification that drops the 7 days immediately around the cutoff yields an enrollment estimate of 0.108 (SE = 0.022), essentially identical to the baseline. Propensity-score reweighting affects point estimates by <5%.

A particularly telling diagnostic is the placebo-cutoff exercise. Across 200 placebo cutoffs drawn uniformly within ±30 days of September 1, excluding a ± 7-day window around the true date, the placebo RDD estimates are on average 0.002 with SD 0·011. The true estimate of 0·112 is about 10 times the standard deviation to the right of the distribution of placebo estimates (Figure 3), which is much greater separation than one would find published RDD work in health-policy applications.

**Figure 3:**
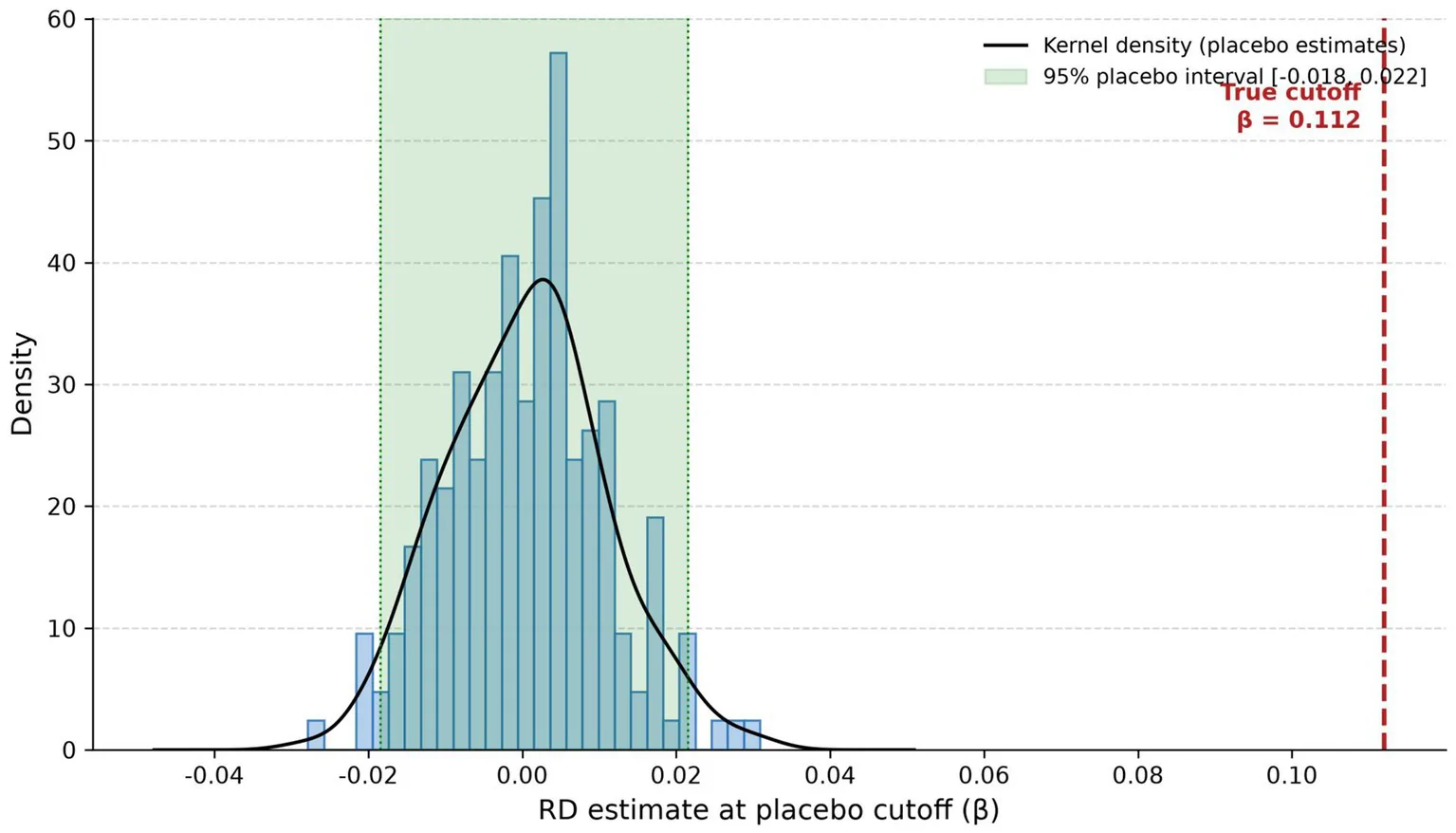
Distribution of RD estimates across 200 placebo cutoffs. Placebo-cutoff distribution. Histogram of RDD enrollment estimates from 200 placebo cutoffs drawn uniformly within ±30 days of true date, excluding ±7-days window. The kernel density is overlaid; the dashed line marks the true estimate at 0.112, which sits far in the right tail. The shaded area denotes the 95% interval of placebo estimates. RDD, regression discontinuity design; SD, standard deviation.

### Heterogeneity analysis

Two clear patterns emerge. First, effects are twice as large for students with rural hukou as for their urban peers (enrollment effect 0.184 vs. 0.089, *p* for difference < 0.01; knowledge effect 0.62 vs. 0.44). Second, non-only children and students in poor or fair self-rated health also show larger gains. Table 3 reports full subgroup estimates on the enrollment outcome. An important caveat applies to the rural–urban subgroup analysis: because the hukou item appeared only on the financial-aid survey module, the subgroup analysis by rural versus urban hukou is restricted to the approximately 60% subsample of students who completed the financial-aid portion of the questionnaire. The observed larger effect for rural students should therefore be interpreted as a pattern within financially vulnerable student groups who applied for aid, rather than an unconditionally representative estimate of rural–urban effect differences for the full student population (Figure 4).

**Table 3:** Heterogeneity in the enrollment effect across subgroups (fuzzy RDD, 45-day CCT bandwidth).

Subgroup	*n*	Pre-cutoff mean	Post-cutoff mean	*β*	SE	*p*
Rural hukou	1,231	0.802	0.986	0.184	0.028	<0.001
Urban hukou	1,680	0.920	0.985	0.089	0.020	<0.001
Only child	1,895	0.892	0.977	0.085	0.019	<0.001
Not only child	2,977	0.861	0.990	0.129	0.024	<0.001
Self-rated health poor/fair	1,043	0.801	0.984	0.183	0.031	<0.001
Self-rated health good/excellent	3,829	0.893	0.986	0.093	0.018	<0.001

RDD, regression discontinuity design; CCT, Calonico–Cattaneo–Titiunik; BMIUR, Basic Medical Insurance for Urban and Rural Residents; KHB, Karlson–Holm–Breen; HIPI, Health Insurance Protection Index; SE, standard error. All models include baseline covariates and use a triangular kernel with a 45-day CCT optimal bandwidth; standard errors are clustered at the major-by-cohort level. Subgroup sample sizes (*n*) refer to observations within the optimal bandwidth. Primary reason for the missing-data mechanism of the household registration (hukou) variable: The baseline questionnaire used in this study was embedded within the “Family Financial Status Collection” module of the university’s new-student onboarding system. According to the workflow of the university’s Student Financial Aid Administration Center, only students who applied for family financial hardship certification, the Green Channel program, student-origin-based student loans, or various need-based grants/encouragement scholarships were routed by the system to the page containing the item on “household registration status (agricultural/non-agricultural).” Students who did not apply for any form of financial aid (approximately 40% of the sample) skipped this question group immediately after completing the basic information section, and the hukou variable was therefore left blank by the system.

**Figure 4:**
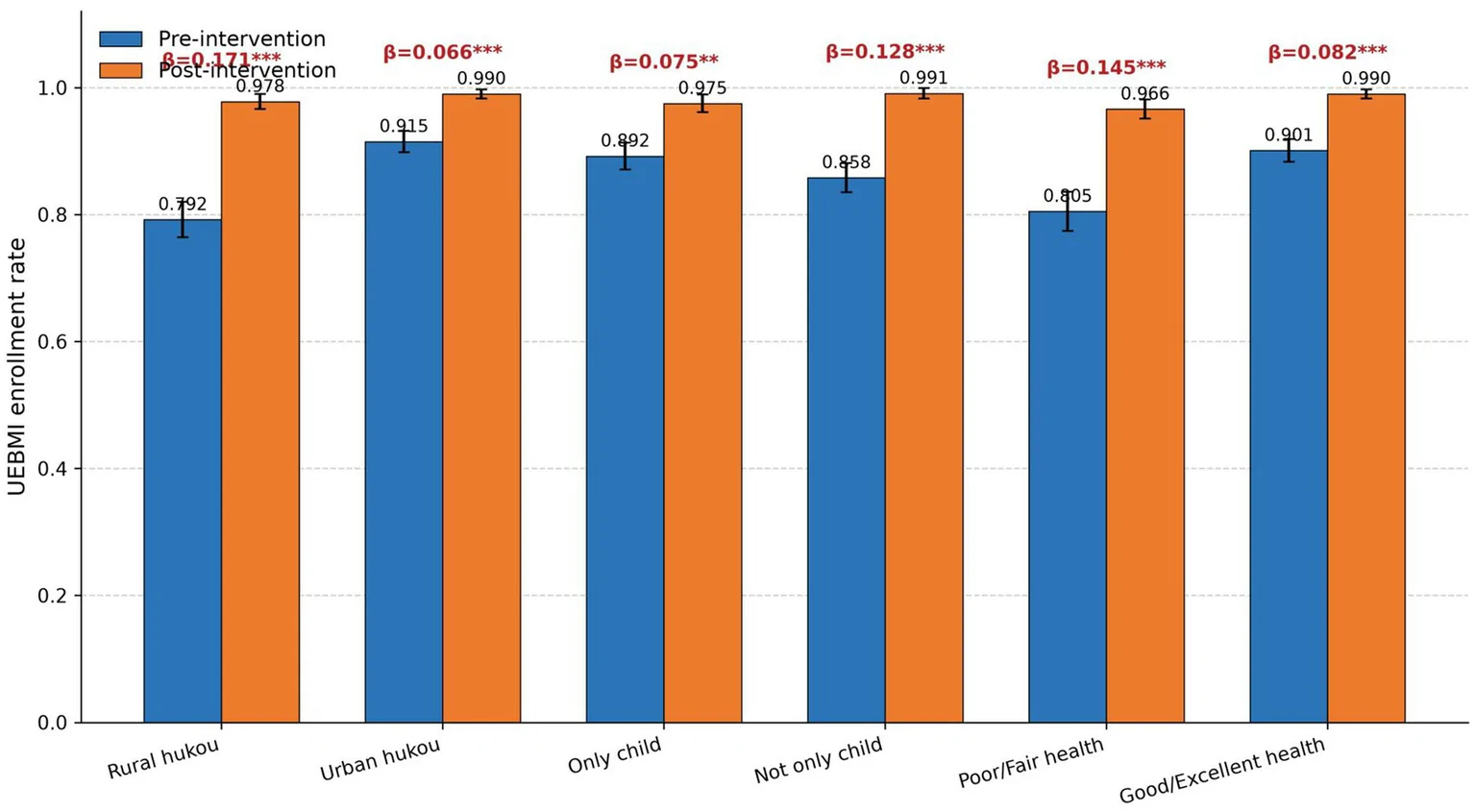
Heterogeneity in the digital health education effect across student subgroups. Heterogeneity in the enrollment effect across six subgroups. Blue bars show pre-cutoff means, orange bars post-cutoff means, with error bars for 95% CIs. Subgroup-specific RD estimates are annotated above each pair (***p < 0.001, **p < 0.01). Rural, non-only-child, and poorer-self-rated-health students show the largest gains. RDD, regression discontinuity design; CCT, Calonico-Cattaneo-Titiunik; SE, standard error.

### Mediation analysis

KHB decomposition attributes 89% of the total effect to the three measured channels. The cognitive channel (knowledge of policy rules) carries the largest share at roughly 47% of the indirect effect; the behavioral channel (platform registration and use of cross-region pathways) accounts for approximately 34%; the psychological channel (trust and perceived usefulness) accounts for about 19%. The direct residual—which captures everything else the module might have done that is not mediated by the three measured channels—is small (11%) and statistically indistinguishable from zero at conventional levels. This non-significant residual should be interpreted with caution: it indicates only that the three measured channels (cognitive, behavioral, and psychological) account for nearly all of the estimated treatment effect in this study’s sample and analytical setting, and does not definitively rule out the existence of other unmeasured mediating pathways that may not have been captured by the study’s survey items. The KHB decomposition is bounded by the mediators we were able to operationalize, and other plausible channels—such as changes in peer effects or in perceived social norms around insurance use—were not directly assessed.

The channel is intuitive. Watching a six-minute explanation of referral rules makes a student more likely to recognize that they need a referral before seeking care off-campus, which directly reduces claim denial rate. Being walked through the one-click activation button on the mini-program sidesteps the paperwork friction that otherwise causes busy freshmen to delay registration. The psychological component (23, 24) is smaller, more consonant with the view that information and activation, rather than attitude change, do most of the work (Figure 5).

**Figure 5:**
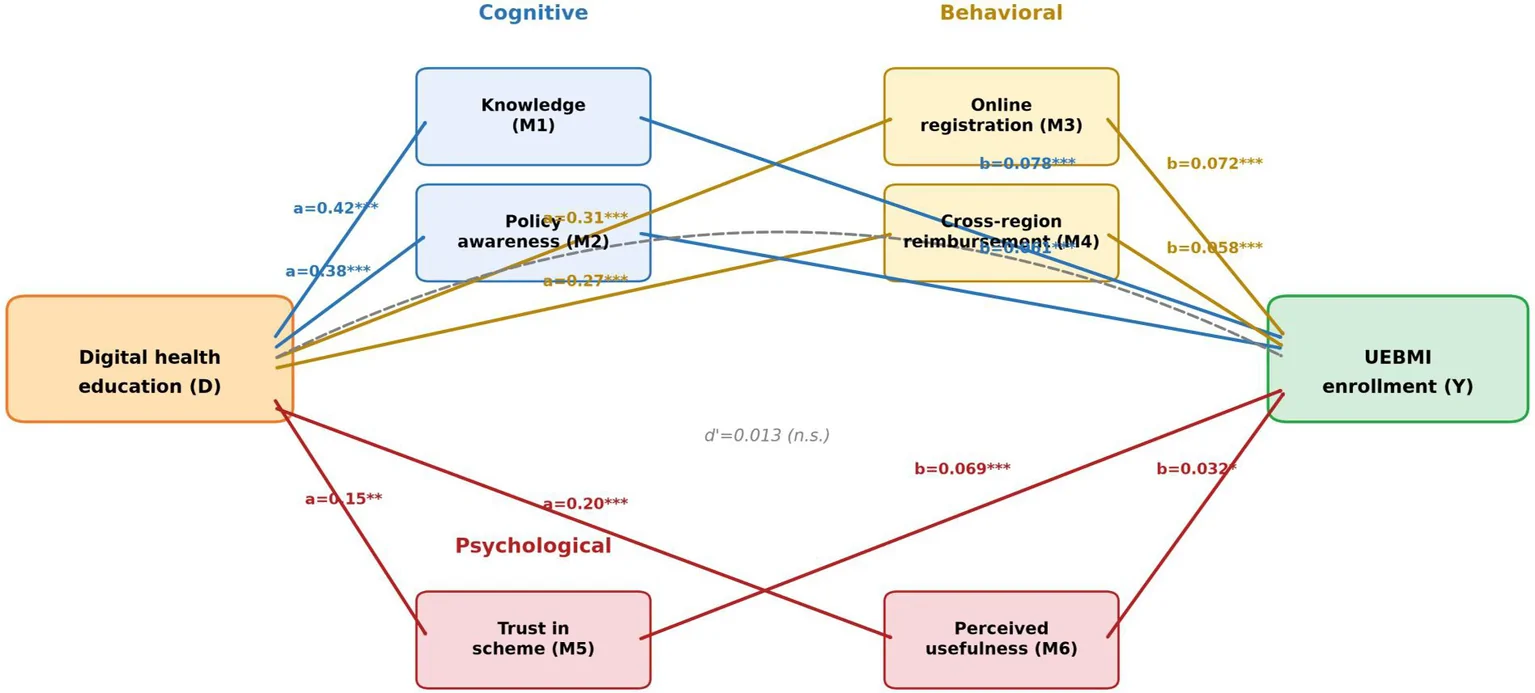
KHB mediation pathways: cognitive, behavioral, and psychological channels. KHB mediation path diagram. D, the intervention (top); Y, the outcome of interest (enrollment; bottom). D affects Y via the cognitive channel (blue), the behavioral channel (yellow) and psychological channel (red). Coefficients a denote effects of D on mediators, b denote effects of mediators on Y conditional on D; ab is the indirect effect for each channel. Direct effect d′ is 0.013 (n.s., dashed); percent of effect is relative share of the total indirect effect. KHB, Karlson-Holm-Breen; HIPI, Health Insurance Protection Index; DHE, digital health education. Indirect: via cognition ab = 0.047***; via behavior ab = 0.033***; via psychology ab = 0.019***. Total effect c = 0.112***. ***p < 0.001, **p < 0.01, *p < 0.05. Solid arrows: significant, dashed gray: non-significant direct effect, KHB decomposition, *n* = 4,872.

## Discussion

Three findings from our study were particularly noteworthy. First, Our RD estimates show a sharp, discontinuous jump in insurance protection outcomes right at the launch of the digital health education program, indicating the intervention is a highly plausible driver of the observed large improvement in outcomes. An 11-percentage-point increase in activation and an 43% relative drop in claim denial rate are not marginal; they translate, at this single university, into several hundred additional students who activate their accounts and dozens fewer out-of-pocket payment incidents per year. Second, the heterogeneity pattern points to where the module adds the most value—among rural students, non-only-children, and students in poorer health—which are precisely the groups that earlier qualitative work identified as most at risk of falling through the cracks (13, 14). Third, mediation suggests the intervention works through cognition and hands-on activation rather than attitudinal change; the implication is that future iterations of such programs should double down on the concrete, procedural parts of the module rather than on generic persuasion about the virtues of insurance.

These findings sit comfortably with a growing literature on nudge-style and digital interventions (19, 22) in social insurance and public benefits. Duflo and co-authors’ work on take-up of social programs has repeatedly found that small logistical frictions are major barriers (20, 21) to enrollment even among eligible populations (15). In the Chinese health-insurance context, several recent studies point to similar patterns among rural-to-urban migrants (16, 17, 30), and among older adult populations (18, 32, 33). Our contribution is to show that the same friction story applies to university students, a population that is sometimes assumed to be digitally native and therefore frictionless in their engagement with online government services. The data suggest otherwise: watching someone else demonstrate a digital tool is rarely a substitute for activating the tool on one’s own phone during a structured, mandated-on-paper module that is coupled to another service the student wants to use (in this case, online health-center booking).

The very sharp drop in claim denial rate is, from a policy perspective, perhaps the most important outcome of all. Claim denial rate in student insurance is, in most cases, not a matter of benefit generosity, it is almost always a matter of procedural mismatches (34): seeing a GP at a non-participating hospital without prior referral, not pre-authorizing an inpatient admission, or not knowing how to ask for cross-region remuneration during the winter break. These are all procedural gaps that a short and focused teaching module (35) is well positioned to address, consistent with the 43% relative reduction in claim denials observed at the cutoff in our RD estimates.

### Implications for policy and practice

Several practical implications follow. First, the cost of the intervention was modest: the total cost of video production, mini-program development, and staff coordination amounted to less than RMB 5 per student, compared with the hundreds of RMB in avoided out-of-pocket costs implied by the reduction in claim denials. Extrapolated to China’s national college and university enrollment of approximately 48 million students, the potential aggregate impact is substantial (26). Second, the one-click integration into a campus platform students already use (in this instance, the WeChat enterprise account) almost certainly is part of what makes this behavioral channel so strong; stand-alone applications or emailed informational brochures (36, 37) are substantially less effective at reducing the activation gap. Third, the module should be revisited annually, not only at the matriculation: renewal of insurance each autumn is yet another decision point where reminder nudges based on the same mini-program can keep the protection level high over the 4 years of enrollment.

### Limitations

This study also has several limitations that require attention. First, the study takes place at one university in Shandong, and the estimated effect is a local average treatment effect (LATE) for compliers at the 2024 enrollment cutoff of this single Shandong public university. Whereas institutional features of student insurance in Shandong share broad structural similarities with student insurance programs across China, several context-specific factors may influence the magnitude of the observed effect: Shandong’s specific 2024 resident health insurance enrollment policy, the high WeChat usage penetration rate among university students in the province, and the close integration between the host university’s campus services and the WeChat enterprise account. The generalisability of these effect sizes to institutions in provinces with different insurance policy rules, lower digital service penetration, or different student demographic profiles therefore requires further multi-site replication. Second, follow-up time was short (3–4 months after intervention), so we cannot yet speak to whether those effects last all the way through sophomore year or even decay; longer-run evidence will matter to institutional decisions whether and how often to repeat the module. Third, survey-based measures of knowledge and satisfaction suffer from reporting bias, though our main enrollment and claim-success outcomes come from records and are not subject to this concern. Fourth, KHB decomposition identifies shares of the mediated effect only under the standard sequential-ignorability assumption; we cannot rule out unmeasured confounders of the association between the mediators and outcome entirely.

## Conclusion

China’s student health insurance scheme has “seen nearly complete uptake by all on paper”, but usable protection at the point of care still leaves many students meaningfully under-insured. This evidence from a Shandong campus—using a well-identified RDD that shows sharp, discontinuous jumps in insurance protection outcomes right at the launch of the digital health education program—indicates that a comparably lightweight digital education intervention, delivered where students already are and packaged around services they already use, is a highly plausible driver of substantial improvements in insurance protection for compliers at the cutoff. The biggest gains accrue to the students who need protection most. This finding is consistent with a well-established principle in behavioral public finance: when the gap between eligibility and uptake is procedural, targeted procedural education—not just more generous benefits—is what gets people from being insured on paper to being protected in practice. Future studies should further this evidence through multi-campus randomized rollouts and longer follow-up windows to establish whether one shot of onboarding is enough, or whether annual booster modules would be required to preserve protection over the whole college cycle.

## Data Availability

The raw data supporting the conclusions of this article will be made available by the authors, without undue reservation.
